# Pneumonia in Sudan: Systematic and Scoping Review of the Literature and Meta-Analysis

**DOI:** 10.7759/cureus.46473

**Published:** 2023-10-04

**Authors:** D M Mohamed, M A SalahEldin, A B Idris, E B Idris, S G Mohamed, Marwan M Badawi

**Affiliations:** 1 Internal Medicine, Sultan Qaboos University Hospital, Muscat, OMN; 2 Medical Microbiology, University of Khartoum, Khartoum, SDN; 3 General Surgery, Sudan Medical Specialization Board, Khartoum, SDN; 4 Medical Microbiology, Rashid Medical Complex, Riyadh, SAU; 5 Medical Unit, Higher Academy for Strategic and Security Studies, Khartoum, SDN

**Keywords:** sub-saharan africa, risk, respiratory, developing countries, africa

## Abstract

In addition to excessive burden of non-communicable diseases, natural and manmade disasters, and internal conﬂicts, Sudan is predominantly susceptible to communicable diseases, such as malaria, tuberculosis, and pneumonia, which bring about an extra burden of demand for high-quality healthcare. According to the WHO and the Sudan Health Observatory, pneumonia is one of the leading causes of death in Sudan. This study therefore aimed to illustrate pneumonia literature in Sudan, estimate infection prevalence regardless of the cause among Sudanese children and adults, and demonstrate its related risk factors. A systematic and scoping review of the literature was conducted and regulated in accordance to the Preferred Reporting Items for Systematic Reviews and Meta-Analyses (PRISMA). After abstract and full-text screening, only 15 articles met our inclusion criteria and passed the quality assessment procedure. Seven included studies determined prevalence of pneumonia; the overall pooled prevalence was around 30%. Furthermore, 12 research articles investigated risk factors related to pneumonia among Sudanese population. Further research with larger sample sizes targeting risk factors of pneumonia among Sudanese population is needed to be conducted.

## Introduction and background

In addition to excessive burden of non-communicable diseases, natural and manmade disasters, and internal conﬂicts, Sudan is predominantly susceptible to communicable diseases, such as malaria, tuberculosis, and pneumonia, which bring about an extra burden of infectious diseases and demand for high-quality healthcare [1].

According to the WHO and Sudan Health Observatory (SHO) in the Sudanese Federal Ministry of Health, the major communicable diseases contributing to morbidity are malaria, schistosomiasis, pneumonia, tuberculosis, and diarrheal diseases [2-4].

Pneumonia is the single largest infectious cause of death in children worldwide. It killed 808,694 children under the age of five in 2017, accounting for 15% of all deaths among children below five years of age. Pneumonia affects children and families everywhere, but it is most prevalent in South Asia and Sub-Saharan Africa. According to the SHO, it was the third cause of death in 2017 among inpatients in Sudan as 6% of the total deaths were due to pneumonia [5-7].

This study therefore aimed to illustrate pneumonia literature in Sudan, estimate infection prevalence regardless of the cause among Sudanese children and adults, and demonstrate its related risk factors. The identification of knowledge gaps will be a giant leap and of great value to the health security of the people of Sudan. 

This article was previously posted to the medRxiv preprint server on September 26, 2022.

## Review

Materials and methods

Search Strategy

To identify relevant studies, a systematic review of the literature was conducted on June 1, 2022. The review was regulated in accordance with the Preferred Reporting Items for Systematic Reviews and Meta-Analyses extension for Scoping Reviews (PRISMA-ScR) [8] (see Appendix A). A comprehensive search was operated in PubMed, Embase, Google Scholar, Scopus, Index Copernicus, Directory of Open Access Journals (DOAJ), EBSCO-Cumulative Index of Nursing and Allied Health (CINAHL), and Cochrane databases without language limits (studies written in languages other than English were later excluded). To obtain a current situation evidence, only studies published in or after 2010 were included. Furthermore, all studies where the data collection process took place before 2010 were also excluded; the only exception was if the collection process started in or before 2010 and ended in 2010 or afterward as previously described [9].

As the medical literature in Sudan is generally scarce in international databases and risk factors may be differently reported, risk factors were not used in keyword formulation, and their related results were later extracted from the included studies. Moreover, studies toward COVID-19 infection were not included as the situation is intended to be evaluated despite of the current COVID-19 pandemic; however, studies concerned with COVID-19 prevalence, not risk factors, were included as it is considered one of the possible causes of pneumonia. Nevertheless, studies investigating COVID-19-associated risk factors without any prevalence determination were cited without their results being scoped to guide readers about the current literature and to illustrate knowledge gaps.

As previously described [10], the keywords used in PubMed were as follows: (Lower respiratory tract infection) OR Pneumonia OR Pneumonias OR (Lung inflammation) OR Lobitis OR (non specific inflammatory lung diseases) OR Prepneumonia OR pleuropneumonia OR Pleuropneumonitis OR (Pneumonic lung) OR (Pneumonic pleurisy) OR (Pneumonic pleuritis) OR Pnemonitides OR Pneumonitis OR (Pulmonal inflammation) OR (Pulmonary inflammation) OR (Pulmonic inflammation) AND Sudan*[tiab].

Moreover, to optimize our search, hand searches of reference lists of the included articles were also performed.

Study Selection and Data Extraction 

Titles and abstracts were assessed for preliminary eligibility. A copy of the full text was obtained for all research articles that were hand-approved in principle to be included. Abstraction of data was in accordance with the task separation method; method and result sections in each study were separately abstracted in different occasions to reduce bias. Moreover, data abstraction was conducted with no consideration of the author’s qualifications or expertise as described in details previously [9]. Each research article was screened for all relevant information and recorded in the data extraction file (Microsoft Excel), data from each method section were extracted using a predefined set of variables: study characteristics, type of the causative organism, type of participants, study population size, geographical region, methodology used in prevalence or risk assessment, and the period of the study conduction.

After inclusion, studies were further classified into studies determining prevalence, studies determining risk factors, and studies determining both prevalence and risk factors. Furthermore, as risk factor-related keywords were not formulated in the search strategy, each study was fully screened to check the nature of the risk investigated.

Whenever outcomes related to specific risk factors are scarce where a meta-analysis cannot be synthesized, scoping of the literature was conducted to highlight methodologies and results conducted and to illustrate research gaps among Sudanese population.

Assessment of Quality

Each included article was evaluated based on a framework for making a summary assessment of the quality. The related published literature was crossed, and then a framework was structured specifically to determine the level of representativeness of the studied population and to judge the strength of the estimates provided. Five questions were to be answered in each article; each answer represent either a score of 1 for yes, 0 for no, or 0 score for not available. A total score for risk of bias and quality was calculated by adding up the scores in all five domains, resulting in a score of between 0 and 5. The highest score indicates the highest quality; only studies with a score for quality greater or equal to 3 (higher quality) were included. As previously described [9], the five domains were as follows: Is the study objective clearly defined? Is the study sample completely determined? Is the study population clearly defined and specified? Is the methodology rigorous? Is the data analysis rigorous?

Quantitative Analysis

A meta-analysis was performed whenever possible using Review Manager software (version 5.3, Cochrane Collaboration, UK). The software automatically provided the confidence interval (CI) according to the calculated standard error (SE). If the CI is provided in a study, it was introduced accordingly. The heterogeneity of each meta-analysis was assessed as well. Although a random effect was favored over the fixed-effect model in all meta-analyses established as variations between studies are predicted to be probable due to the diversity of the study populations, both random and fixed-effect models were investigated. Sensitivity analysis was also approached, whenever indicated, to determine the effect of studies conducted in populations proposed to behave in indifference manners or proposed to be more health-educated on the overall pooled prevalence. Moreover, a subgroup analysis was conducted whenever suitable to determine the prevalence or risk level in a specific state or population. An outcome to take part in the meta-analysis has to be included in at least two studies.

The trim-and-fill method was used to assess the risk of publication bias in each meta-analysis conducted [11].

Results

Studies Included

**Figure 1:**
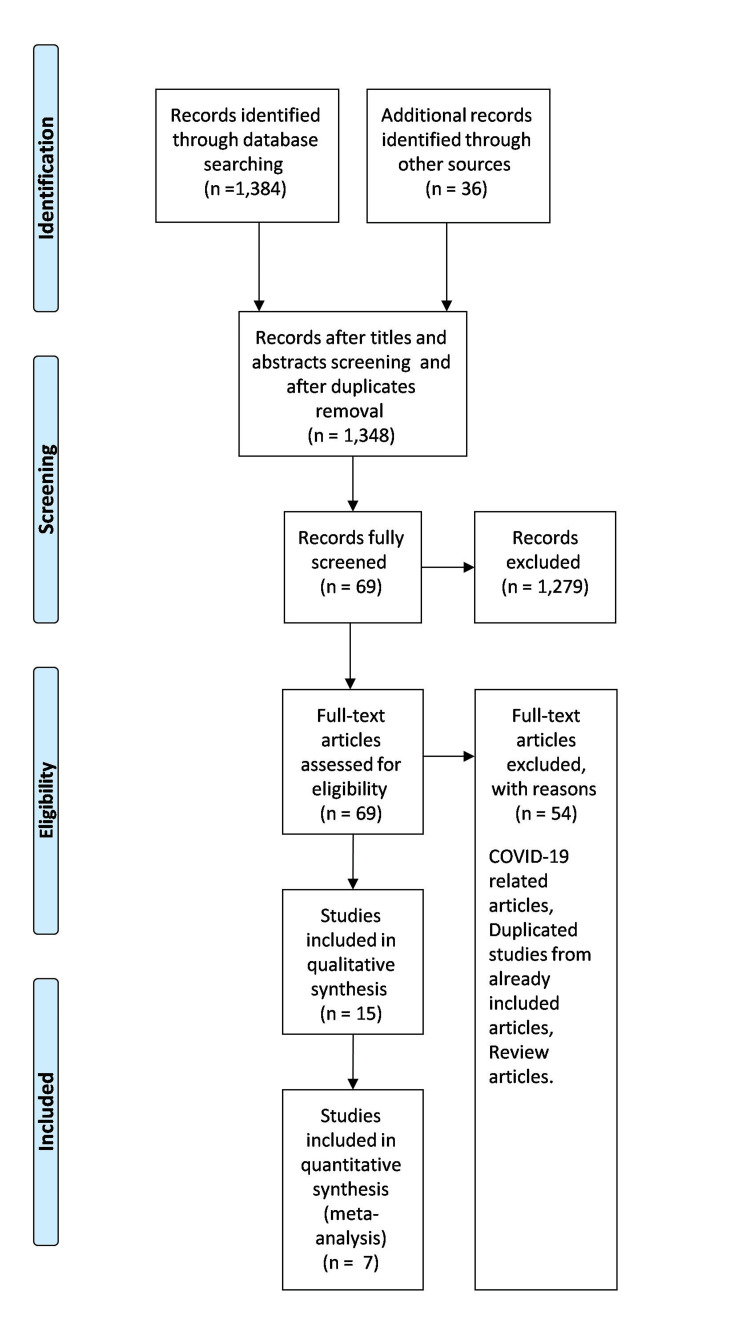
Preferred Reporting Items for Systematic Reviews and Meta-Analyses (PRISMA) flow diagram

A total of 1,420 articles were identified from the search strategy, including hand searches of reference lists of included original research articles and reviews. From these, 1,348 articles were excluded. After abstract and full-text screening, only 15 articles met our inclusion criteria and passed the quality assessment procedure [12-26]. The articles reported prevalence among specific population and/or risk factors. Figure 1 illustrates the PRISMA flow diagram. The included articles are depicted in Table 1. The assessment of the quality of included studies is depicted in Appendix B.

Study Characteristics

The characteristics of the included studies are depicted in Table 1, among which the oldest was published in 2011 [23] while the most recent one was published in 2022 [20]. Twelve research articles determining pneumonia prevalence or risk factors were conducted in Khartoum State [12,14-17,20-26], two in Gezira State [18,19], and one in South Darfur State [13]. Moreover, almost all articles were conducted among both genders, but one study recruited females only (mothers of infected children) [18], while another study did not determine the gender of their participants [17]. Furthermore, 10 articles were concerned of pneumonia prevalence/risk factors among children [14,15,17-19,22-26]. All characteristics of the included studies are depicted in Table 1. The publication bias assessment indicated no major asymmetry.

**Table 1:** Characteristics of the included studies

Study ID	Year of publication	Study design	City/state	Study population/s	Assessment	Sample size	Gender	Participants' age
Abd Elrhman et al. [12]	2015	Cross-sectional	Khartoum State	Suspected patients	Prevalence of *Moraxella catarrhalis* (bacteriological methods)	200	Both	19-86 years
Essa et al. [13]	2017	Retrospective	South Darfur State	Suspected patients	Prevalence	9,000	Both	1-44 years
Gabbad et al. [14]	2014	Cross-sectional	Khartoum State	Children	Prevalence of *Streptococcus pneumoniae* (bacteriological methods) and risk factor	282	Both	<5 years
Gritly et al. [15]	2018	Cross-sectional	Khartoum State	Children	Risk factors	40	Both	<5 years
Idress [16]	2017	Cross-sectional	Khartoum State	Nurses	Risk factors	60	Both	20 to >40 years
Mahgoub et al. [17]	2013	Cross-sectional	Khartoum State	Children	Prevalence and risk factors	260	Not determined	Not determined
Mohammed [18]	2013	Cross-sectional	Gezira State	Mothers of children less than five years	Risk factors	250	Female	18-63 years
Mohamoud [19]	2014	Cross-sectional	Gezira State	Parents of children less than 5 years	Risk factors	182	Both	20 to >50 years
Moser et al. [20]	2022	Cross-sectional	Khartoum State	General population	Prevalence of SARS-Cov-2 (ELISA) and risk factor	2,374	Not determined	<5 to ≥50 years
Omar et al. [21]	2016	Cross-sectional	Kassala City	Pulmonary TB patients	Prevalence of* S. pneumoniae* (bacteriological methods)	150	Both	10-50 years
Salah et al. [22]	2015	Cross-sectional	Khartoum State	Children with pneumonia	Risk factors	150	Both	2-60 months
Salih et al. [23]	2011	Cross-sectional	Khartoum State	Children with severe pneumonia	Risk factors	224	Both	0-59 months
Salih et al. [24]	2014	Cross-sectional	Khartoum State	Children with severe pneumonia	Risk factors	208	Both	Mean = 28 months
Salih et al. [25]	2015	Cross-sectional	Khartoum State	Children with severe pneumonia	Risk factors	195	Both	2-59 months
Salih et al. [26]	2015	Cross-sectional	Khartoum State	Children with severe pneumonia symptoms	Prevalence (bacteriological methods)	189	Both	<5 years

COVID 19-Related Studies

Studies concerned with pathogenesis, health services, knowledge, behaviors, or risks related to COVID-19 were beyond the scope of this review. Forty-one studies were found to be addressing COVID-19 among the Sudanese population [27-65] according to the current search strategy.

Prevalence of Pneumonia

Out of 15 included studies, seven studies determined the prevalence of pneumonia, three studies determined the prevalence among suspected outpatients, one study determined the prevalence among tuberculosis patients, while the other three studies determined pneumonia prevalence among children. Furthermore, three studies determined the prevalence of several microorganisms causing pneumonia, two studies were concerned with the prevalence of *Streptococcus pneumoniae*, one was concerned with the prevalence of *Moraxella catarrhalis,* and one was concerned with the prevalence of SARS-Cov-2. The characteristics of the studies are depicted in Table 1.

**Figure 2:**
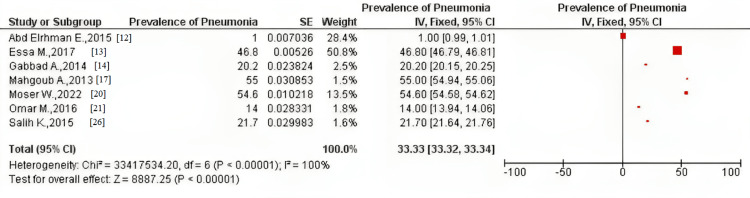
Pooled prevalence of pneumonia among the included studies using the fixed-effect model

**Figure 3:**
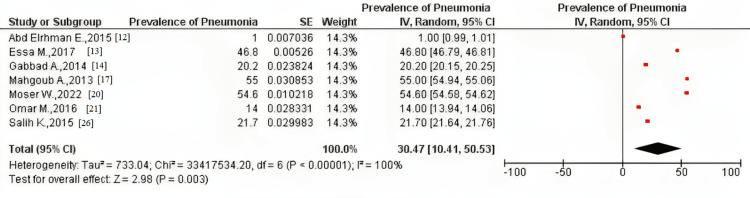
Pooled prevalence of pneumonia among the included studies using the random-effect model

The pooled prevalence of all seven studies using the fixed-effect model was 33.33% (CI = 33.32, 33.34) and 30.47% (CI = 10.41, 50.53) using the random-effect model. Moreover, after conducting a sensitivity analysis, the fixed-effect pooled prevalence was 30.02% (CI = 30.01, 30.03) and 26.45% (CI = 3.16, 49.74) using the random-effect model. Heterogeneity was high among all the meta-analyses conducted (I^2^=100%) (Figures 2, 3).

**Figure 4:**
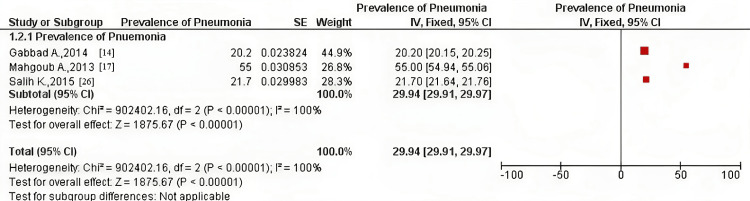
Pooled prevalence of pneumonia in children among the included studies using the fixed-effect model

**Figure 5:**
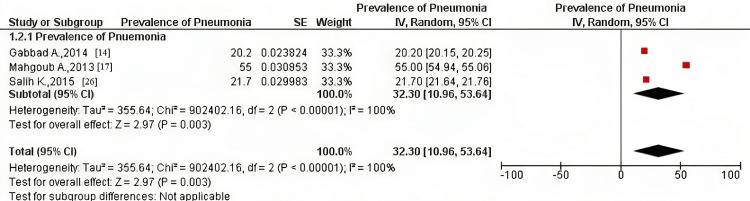
Pooled prevalence of pneumonia in children among the included studies using the random-effect model

The pneumonia prevalence of children (below five years of age) was investigated among three included studies [14,17,25], whose participants were all outpatient children from both genders in a study [14] and suspected outpatients from both genders in the remaining two studies [17,25]. Pooled prevalence using the fixed-effect model was 29.94% (CI = 29.91, 29.97) and 32.30% (CI = 10.96, 53.64) using the random-effect model (Figures 4, 5).

Risk Factors

Five research articles investigated risk factors related to pneumonia among the Sudanese population.

The study of Gritly et al. [15] was conducted to determine the risk factors contributing to pneumonia at Omdurman City, Khartoum State, among 40 children under five years of age using a questionnaire; 57.50% were males and less than one year old, while 67.50% of the patients' fathers were laborers (workers), 55.0% had a basic education level, and 65.0% make less than 150 *SDG* (per day). Moreover, most of the children (72.50%) were breastfed. Majority of the study participants (65.0%) used tap water, and most of the study participants (mothers) (47.50%) used a zeer pot fridge for drinking water and storage. No determination of any significant associations was described in the study.

Moreover, the study of Salih et al. [23] investigated 224 children under five years of age who presented to the emergency unit at Gaafar Ibn Oaf Children’s Hospital and Omdurman Children’s Hospital, Khartoum State, and were assessed and managed for severe presentation of pneumonia. The participants were 52.7% female, 41.0% were less than one year of age, 29.0% were breastfed for 13-18 months, 65% had unemployed parents, 67.9% were using gas for cooking, and 67.4% have their fathers as a source of exposure to tobacco smoke. No determination of any significant associations was described.

Another study [14] measured the proportion of pneumonia among 282 children attending Omdurman Paediatric Hospital, Khartoum State, to highlight disease-related factors, using a questionnaire. Their results indicated that male gender (odds ratio, OR = 2.41), mother illiteracy (OR = 7.54), low income (OR = 1.55), and urban residence (OR =2.09) are sociocultural risks that are significantly related to pneumonia in children.

Furthermore, the study of Mahgoub et al. [17] aimed to determine the prevalence of pneumonia among children under five years of age in Khartoum State and to investigate associated factors using a questionnaire. Four hundred families were included in their study. Income (OR = 2.73), mother's education less than the university level (OR = 1.4), and house size (one to two rooms; OR = 2.36) were concluded as sociocultural risks that are significantly related to pneumonia in children.

Lastly, the study of Salih et al. [25] aimed to determine the risk factors of death among 195 children aged 2-59 months hospitalized for severe pneumonia in Khartoum State. The authors investigated the significant change of outcome (recovery vs. death) according to gender. Their results indicated that males are more likely to die (OR = 1.1), but a significant difference was not concluded.

Knowledge, Practice, and Awareness Assessment

Three included research articles investigated knowledge, practice, or awareness related to pneumonia among the Sudanese population.

One study [16] was aimed to assess nurses' knowledge and practice regarding the nursing care of patients with ventilator-associated pneumonia using an interview questionnaire at Ahmed Gasim Hospital, Khartoum State. Sixty nurses were recruited; only 34.5% of the participants responded correctly regarding signs of ventilator-associated pneumonia and prevention methods of ventilator-associated pneumonia. Moreover, 85% correctly practiced gastric reflux prevention, 55% practiced equipment maintenance, 73.69% practiced suctioning from the endotracheal tube (ETT)/tracheotomy, and 68.65% practiced care of patients with ventilator-associated pneumonia.

Another study [18] aimed to assess mothers’ knowledge and recognition of pneumonia among children less than five years of age in Bashir Banaga Village, Hassahisa locality, Gezira State, and mothers’ attitude toward seeking medical help if they had a child with symptoms of pneumonia. A total of 250 mothers were interviewed using a questionnaire. Some (41.8%) of the participants were 31-35 years old, and 73.2% were unemployed. Weather change (17.6%) and cold temperature (12.4%) were indicated as causes. Only 26% of the mothers reported that a virus or germ causes pneumonia. Moreover, only 23.6% of the mothers said that they would recognize pneumonia if their child had rapid breathing or if their child had cough (34.0%).

Furthermore, the study of Mohamoud [19] aimed to investigate the knowledge, attitude, and practice of 182 parents of children less than five years of age suffering from pneumonia in Daraga district, Wad Madani town, Gezira State, using a questionnaire. A proportion (81.4%) of the parents were 30-49 years of age, 70.3% were still married, 38.5% have secondary education as their education level, and 44.0% were housewives. Moreover, 70.3% of the parents said that a virus causes pneumonia in children, 23.1% said bacteria, 2.2% listed congenital, 2.7% listed parasites, and 1.6% said others. Moreover, 31.3% of the parents listed fever and cough as symptoms of pneumonia, 13.7% listed chest pain, 3.3% listed vomiting, 51.1% listed fever cough and chest pain, and 0.5% listed others. As routes of transmission, 28.0% of the parents said coughing, 31.9% said sneezing, 1.6% said handshakes, and only 37.4% indicated all of them. As the prevention strategy, 59.9% of the parents listed vaccination, good health, and complete cycle of breastfeeding to the child as best ways to prevent childhood pneumonia, and lastly, most parents (73.6%) indicated that they had no animals’ shelter in their houses.

Other Outcomes

The study of Salah et al. [22] aimed to determine the prevalence of hypoxemia among children less than five years old with pneumonia who presented with acute history of cough and rapid respiration or difficulty in breathing in Omdurman City, Khartoum State. Out of 150 children who participated, 57.3% were males, 32% were aged 2 to ≤12 months. A proportion (42.7%) of the patients had hypoxemia (with pulse oximeter oxygen saturation <90%), and 36 (56.25%) were in the age group <2 months. Of the hypoxic patients, 30 (46.88%) had severe pneumonia, and seven (10.94) had very severe pneumonia. Moreover, hypoxemia increased in the male sex but not of statistical significance (P = 0.72), while hypoxemia was significantly increased in patients diagnosed with very severe pneumonia (P < 0.001).

The study of Salih et al. [24] was designed to investigate the adherence and response to the WHO guidelines for the treatment of severe pneumonia in hospitals of Khartoum State. A total of 208 children aged 2-59 months with definite diagnosis of severe pneumonia were included. The mean (standard deviation (SD)) age was reported as 28.12 (13.9) months, and 52.4% were females. Only 39 (18.8%) of the children received the prescription that was adherent to the WHO guidelines for severe pneumonia (penicillin). The antimicrobial prescriptions, which were not in accordance with the WHO guidelines, were amoxicillin/clavulanic acid (46, 22.1%), ceftriaxone (42, 20.2%), cefuroxime (41, 19.7%), a combination of penicillin/gentamicin (29; 13.9%), and others (11, 5.3%). None of the investigated factors (age, gender, symptoms, and signs) were found predictors for adherence to the WHO guidelines for the treatment of severe pneumonia. Moreover, there was no significant difference in the responses between adherent and non-adherent prescriptions. Lastly, none of the investigated predictors was found to be associated with the treatment outcome.

Discussion

To our knowledge, this review is the first attempt to find out the magnitude of information on the pooled prevalence of pneumonia and its associated risk factors in Sudan. A widespread search from several published databases and stringent methodology to screen and include every potential study was approached in the present study.

The pooled prevalence of pneumonia among Sudanese children was around 30% considering the differences between fixed- and random-effect models, with lower estimates reported in Uganda (25%) and Kenya (21%). However, an almost similar result has been reported in the neighboring country Ethiopia (33%) [66,67].

Several included studies assessed sociocultural risk factors related to the acquisition of pneumonia, with male gender, illiteracy, low income, and urban residence as the main sociocultural related risk factors. This finding aligns with several reports in the literature [68-70].

Moreover, a study conducted in Uganda [67] indicated that children of rural residence had 5.7 higher odds of having pneumonia compared to those of urban residence. Rural residence reported in Uganda's finding (OR = 5.7, 95% CI = 2.97-11.05, p < 0.001) is comparable with a study conducted in Ethiopia [71], which reported 4.5 higher odds of developing pneumonia among children of rural residence. Nevertheless, due to exposure to factors, such as air pollution and overcrowding in urban areas, other studies indicated that the odds are higher in urban areas compared to rural areas. Further research is needed to investigate specific ecological risk factors to better study the urban/rural pneumonia infection patterns [72].

Furthermore, it was concluded that the incidence of pneumonia is higher in people at the extremes of age and people living in socially deprived areas as indicated by several included studies. This finding is in agreement with several research articles in the literature [73,74].

Regarding the socioeconomic status and supporting findings scoped from the included studies, a study conducted by Park in 2007 reported that children with a low socioeconomic status tend to have more risk of respiratory infections and that children of mothers with a personal source of income are at a lower risk of developing pneumonia, this is also indicated by a study conducted in the Gambia by O’Dempsey et al. [5,75-77].

Furthermore, only 43.5% of nurses in the included study of Idress [16] correctly responded regarding signs and prevention methods of ventilator-associated pneumonia. This finding is similar to what had been reported in the USA by a study of Torres et al. [77] in 2013 where the average knowledge score regarding signs and symptoms of ventilator-associated pneumonia was 43.28% among the participants. Moreover, poor knowledge was reported as 65% in Sudan in a study by El-Khatib and colleagues in 2010 [78,79].

This review identified several key gaps within the existing body of literature. Studies concerning pneumonia in Sudan are generally scarce that a meta-analysis cannot be synthesized. For instance, only 15 studies were found in the literature that addressed pneumonia prevalence/risk factors, while 41 studies were found to be related to the recent COVID-19 pandemic. Moreover, no studies, to our knowledge, were conducted to investigate specific ecological risk factors toward pneumonia rather than “rural residence.” The risk of pneumonia is partially driven by host genetics, such as the *CYP1A1 *gene. However, no studies, to our knowledge, were conducted to investigate related genetic variations of infected participants and its possible relations to pneumonia or its severity among the Sudanese population.

The strengths of this review are that we systematically identified and included related studies from 2010 to 2022. Moreover, we have conducted a meta-analysis to derive the pooled prevalence estimates of related studies. We also carried out a quality assessment of the included studies based on criteria specifically developed to determine the quality of included studies.

Nevertheless, several limitations are to be considered when interpreting study results. First, gray literature evidence was not assessed. Moreover, African journals that are not indexed in the screened databases were not included. Although all included studies are of good quality, several good studies might have been missed. Furthermore, the included studies used in the current study to determine pooled prevalence estimates adopted different approaches toward prevalence determination. Lastly, the heterogeneity was high among the meta-analysis conducted.

## Conclusions

It is concluded from the current study that pneumonia research is generally scarce among the Sudanese population. The current study findings, according to the included studies, indicate that the pooled prevalence of pneumonia is around 30% among the general population and children.

The health authorities and other related stakeholders need to support research investigating specific ecological and genetic factor associations toward the acquisition of pneumonia, especially among children, as robust knowledge on pneumonia-related risk factors may enhance containment attempts through vaccination or other available means.

Further research with larger sample sizes targeting the prevalence and risk factors of pneumonia among the Sudanese population is needed to be conducted.

## Appendices

Appendix A 

**Table 2:** Preferred Reporting Items for Systematic reviews and Meta-Analyses extension for Scoping Reviews (PRISMA-ScR) checklist

Section	Item	PRISMA-ScR Checklist item	Reported on page #
TITLE			
Title	1	Identify the report as a scoping review.	1
ABSTRACT			
Structured summary	2	Provide a structured summary that includes (as applicable): background, objectives, eligibility criteria, sources of evidence, charting methods, results, and conclusions that relate to the review questions and objectives.	1
INTRODUCTION			
Rationale	3	Describe the rationale for the review in the context of what is already known. Explain why the review questions/objectives lend themselves to a scoping review approach.	2
Objectives	4	Provide an explicit statement of the questions and objectives being addressed with reference to their key elements (e.g., population or participants, concepts, and context) or other relevant key elements used to conceptualize the review questions and/or objectives.	2
METHODS			
Protocol and registration	5	Indicate whether a review protocol exists; state if and where it can be accessed (e.g., a Web address); and if available, provide registration information, including the registration number.	NA
Eligibility criteria	6	Specify characteristics of the sources of evidence used as eligibility criteria (e.g., years considered, language, and publication status), and provide a rationale.	3
Information sources*	7	Describe all information sources in the search (e.g., databases with dates of coverage and contact with authors to identify additional sources), as well as the date the most recent search was executed.	3
Search	8	Present the full electronic search strategy for at least 1 database, including any limits used, such that it could be repeated.	4
Selection of sources of evidence†	9	State the process for selecting sources of evidence (i.e., screening and eligibility) included in the scoping review.	4
Data charting process‡	10	Describe the methods of charting data from the included sources of evidence (e.g., calibrated forms or forms that have been tested by the team before their use, and whether data charting was done independently or in duplicate) and any processes for obtaining and confirming data from investigators.	4
Data items	11	List and define all variables for which data were sought and any assumptions and simplifications made.	4
Critical appraisal of individual sources of evidence§	12	If done, provide a rationale for conducting a critical appraisal of included sources of evidence; describe the methods used and how this information was used in any data synthesis (if appropriate).	4
Synthesis of results	13	Describe the methods of handling and summarizing the data that were charted.	5
RESULTS			
Selection of sources of evidence	14	Give numbers of sources of evidence screened, assessed for eligibility, and included in the review, with reasons for exclusions at each stage, ideally using a flow diagram.	6
Characteristics of sources of evidence	15	For each source of evidence, present characteristics for which data were charted and provide the citations.	7
Critical appraisal within sources of evidence	16	If done, present data on critical appraisal of included sources of evidence (see item 12).	8
Results of individual sources of evidence	17	For each included source of evidence, present the relevant data that were charted that relate to the review questions and objectives.	8
Synthesis of results	18	Summarize and/or present the charting results as they relate to the review questions and objectives.	10
DISCUSSION			
Summary of evidence	19	Summarize the main results (including an overview of concepts, themes, and types of evidence available), link to the review questions and objectives, and consider the relevance to key groups.	16
Limitations	20	Discuss the limitations of the scoping review process.	18
Conclusions	21	Provide a general interpretation of the results with respect to the review questions and objectives, as well as potential implications and/or next steps.	19
FUNDING			
Funding	22	Describe sources of funding for the included sources of evidence, as well as sources of funding for the scoping review. Describe the role of the funders of the scoping review.	N/A

Appendix B 

**Table 3:** Quality assessment of the included studies

Study ID	Is the study objective clearly defined?	Is the study sample completely determined?	Is the study population clearly defined and specified?	Is the methodology rigorous?	Is the data analysis rigorous?	Final score
Abd Elrhman et al., 2015 [12]	1	1	1	1	0	4
Essa et al., 2017 [13]	1	1	1	1	0	4
Gabbad et al., 2014 [14]	1	1	1	1	1	5
Gritly et al., 2018 [15]	1	1	1	1	1	5
Idress, 2017 [16]	1	1	1	1	1	5
Mahgoub et al., 2013 [17]	1	1	1	1	1	5
Mohammed, 2013 [18]	1	1	1	1	1	5
Mohamoud, 2014 [19]	1	1	1	1	1	5
Moser et al., 2022 [20]	1	1	1	1	1	5
Omar et al., 2016 [21]	1	1	1	1	1	5
Salah et al., 2015 [22]	1	1	1	1	1	5
Salih et al., 2011 [23]	1	1	1	1	1	5
Salih et al., 2014 [24]	1	1	1	1	1	5
Salih et al., 2015 [25]	1	1	1	1	1	5
Salih et al., 2015 [26]	1	1	1	1	1	5
